# An Alternative Method of Tensioning Traction Within Thomas Splint

**DOI:** 10.7759/cureus.18304

**Published:** 2021-09-26

**Authors:** Timothy D Pearkes, Matthew Towner

**Affiliations:** 1 Orthopaedic Surgery, North Bristol National Health Service Trust, Bristol, GBR

**Keywords:** telescopic thomas' splint, thomas' splint, femoral fracture, traction, pulley

## Abstract

The application of a Thomas splint when managing a femoral fracture has the potential to be a painful experience for the patient. If movement of the injured limb can be reduced during the application then the patient will likely suffer less pain. In this report, we describe a method that enables the clinician to remove any slack in the tensioning system and apply the traction in a single movement. No additional equipment is required beyond the standard splint and skin traction apparatus. A pulley system is created using the cord, the splint and two overhand knots, minimising movement at the injury site whilst permitting sufficient traction to be applied. Once applied, it can be easily re-tensioned as the thigh musculature relaxes. We believe this method to be simple, more adaptable and quicker to apply than the current standard.

## Introduction

The 'Thomas splint', introduced by Hugh Owen Thomas in 1865, was first used for the treatment of tuberculosis of the knee [1]. During the First World War, its use significantly reduced the battlefield mortality rate associated with open femoral fractures from 80% to 15.8% [2]. The Thomas splint remains in frequent use, predominantly as a temporary treatment prior to surgery, and less frequently as a definitive treatment for a femoral fracture [3,4].

The benefits of early splinting in femoral fractures have been re-affirmed in a recent study. When comparing the pre-hospital application of the Thomas splint with the application after a radiographical diagnosis, the chance of requiring a blood transfusion was halved, and the risk of pulmonary complications fell from 12% to 0% [5].

Traditional splinting involves the application of a skin traction. An appropriately sized, telescopic Thomas splint is applied with padding to the perineum. Slings sequentially elevate the thigh and leg with a slight bolster behind the knee. For transit, traction can be applied through the splint, opposed against the perineum, and with the cords from the stirrup tensioned distally using a windlass. When the patient has been transferred to a bed, the pressure on the perineum can be relieved by converting the splint from transit to static mode using weighted or balanced traction [6].

There is rationale behind all of the elements during the application of the splint. Longitudinal elevation of the leg will flex the hip and de-tension the Iliopsoas tendon. It will also lift the posterior thigh/buttock off the padded hoop and reduce the risk of pressure-related soft tissue injury. Flexion of the knee will de-tension the hamstring and gastrocnemius muscles, both of which cross the joint. This may aid in reducing any muscular spasm causing overlap or shortening of the fracture. It may prevent posterior displacement of the distal femur and will improve rotational control of the leg distal to the fracture.

## Technical report

**Figure 1:**
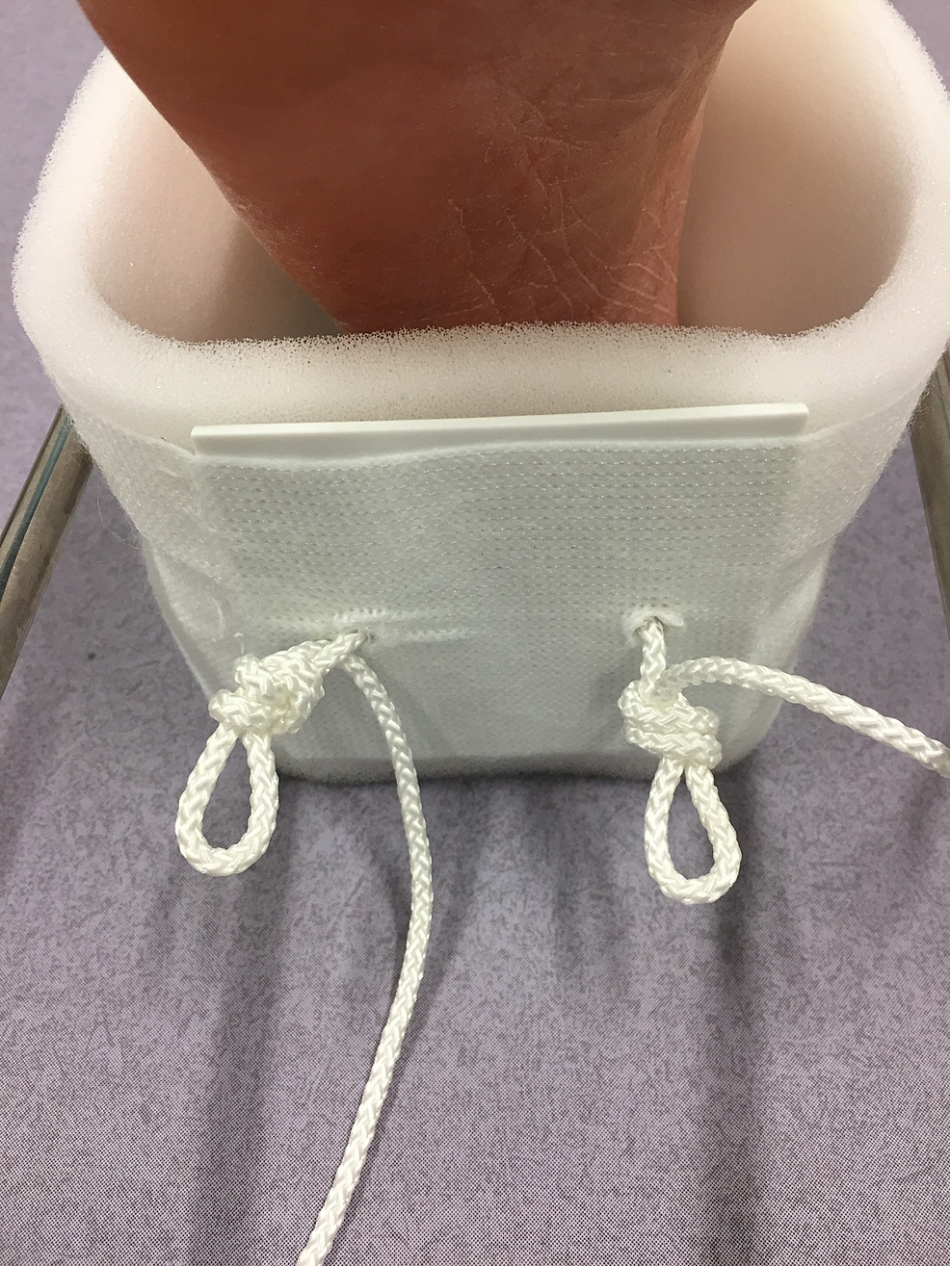
Tying the overhand loop knots

**Figure 2:**
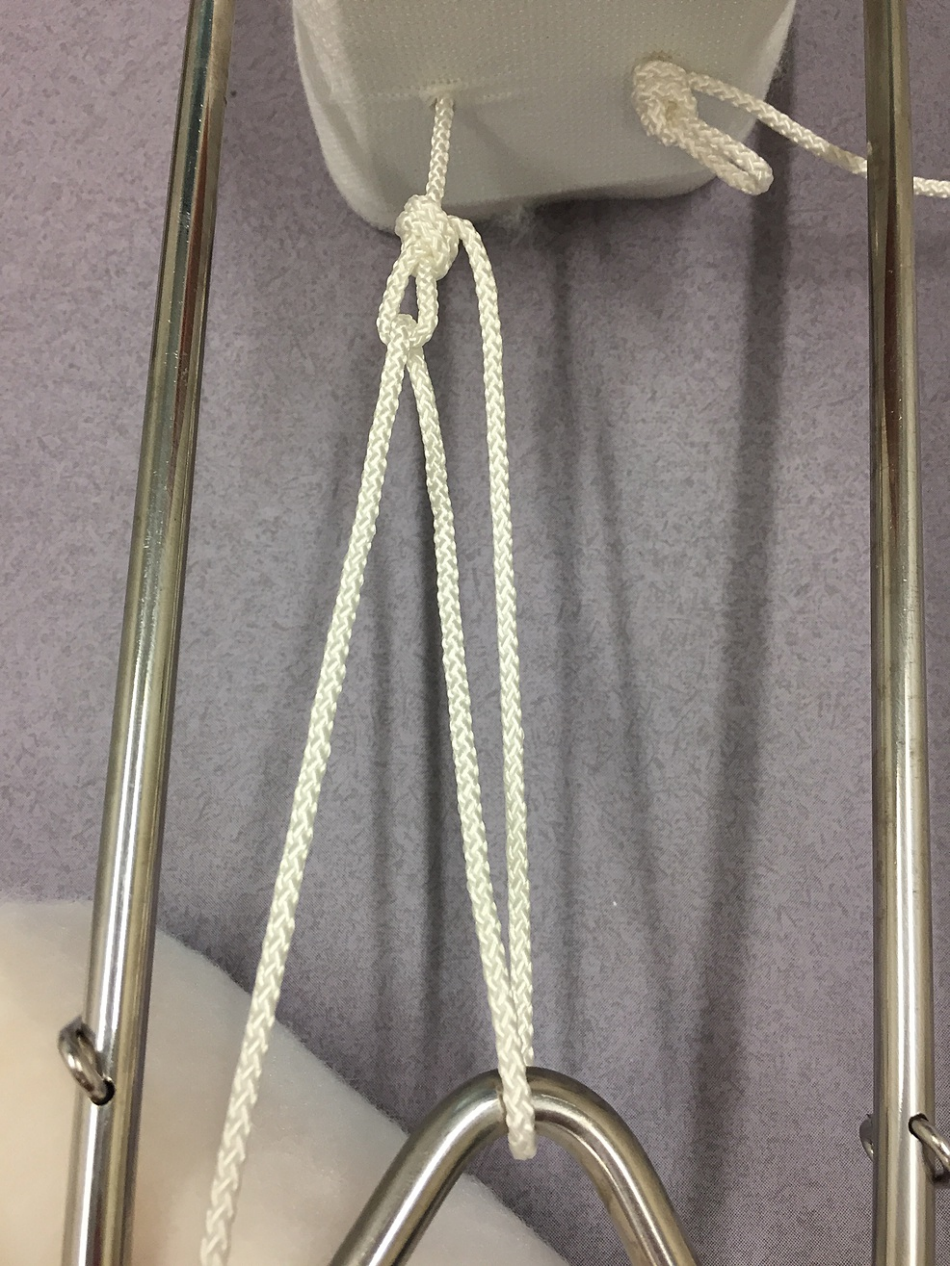
Passing the cord and forming a pulley

**Figure 3:**
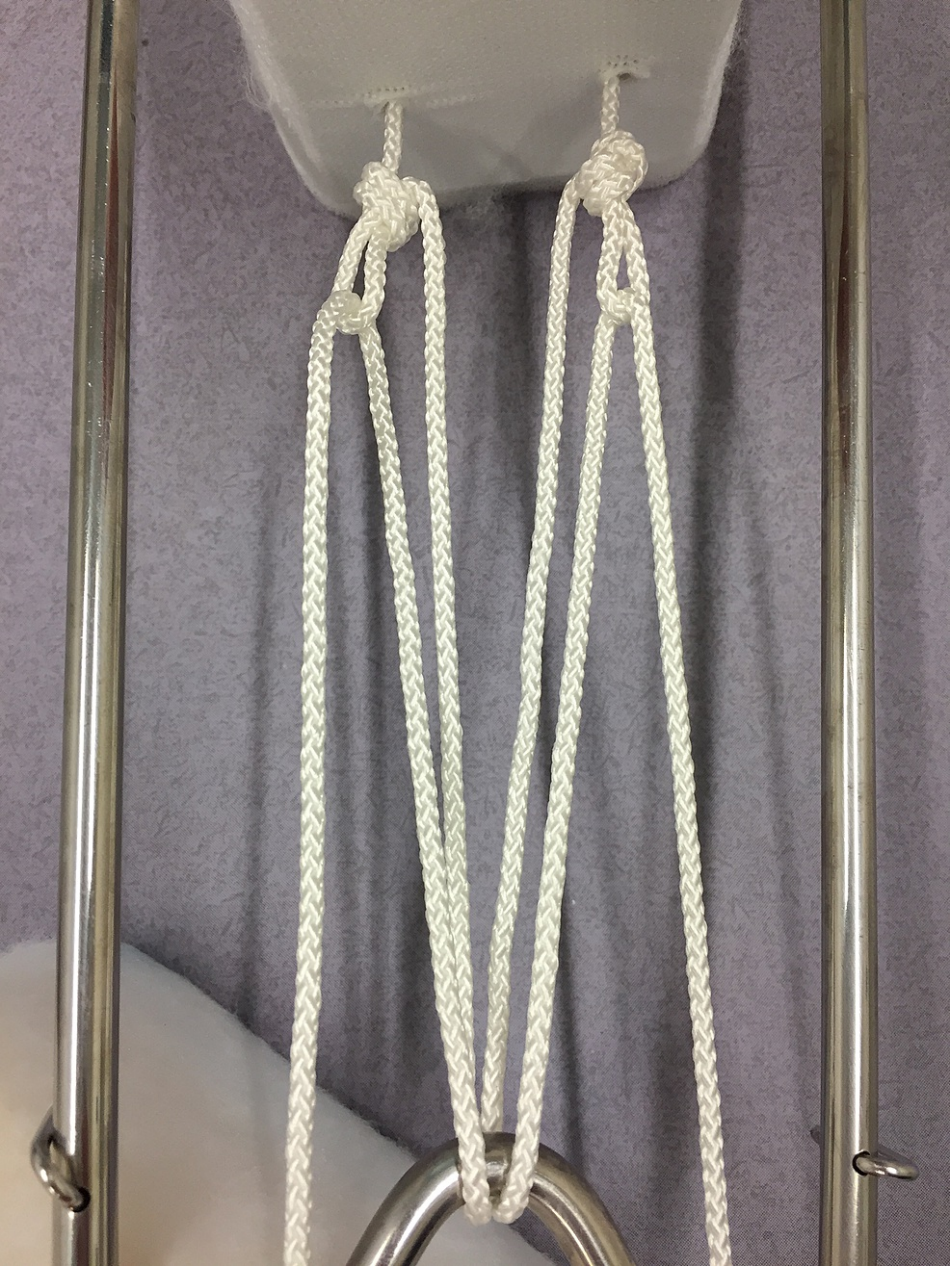
The pulley construct

**Figure 4:**
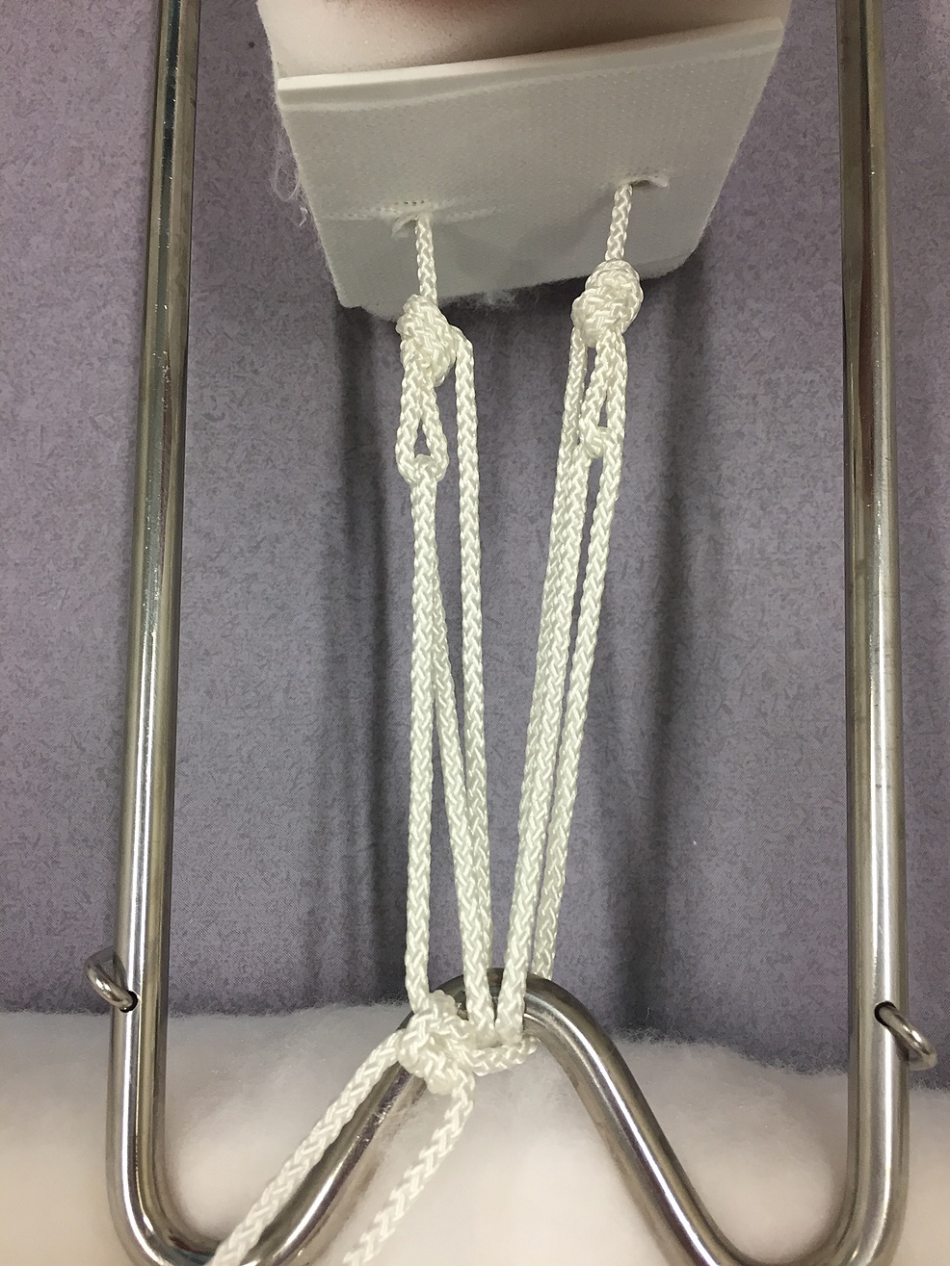
Pulley system completed, locked off with a reef knot

The splint, padding and slings are sized and prepared in the conventional manner [6]. Two non-slip overhand loop knots [7] are tied in the cord as it exits the plastic foot plate of the traction stirrup (Figure 1). The stirrup is then applied and secured with a stretch bandage. The leg is lifted, the splint positioned (with care for the perineum) and the leg rested onto the splint. The two cords are passed around the end of the splint, back through their respective loops, and returned once again to the end of the splint (Figures 2, 3). Traction is applied through both cords, and they are tied to each other under tension around the end of the splint (Figure 4). This final knot can be definitive with a reef knot [8] or temporary with a shoelace bow [9].

With friction eliminated, this pulley will theoretically provide a 3:1 mechanical advantage. For every 3 cm of cord pulled longitudinally, it will apply a further 1 cm of traction. When 1 N of force is applied to the free cords it will achieve 3 N of force in traction. 

## Discussion

The application of a Thomas splint can be painful for the patient. To help tolerate the procedure, the patient should be administered appropriate analgesia. The splint should be fully prepared in advance to ensure no delay in application once it is commenced. 

**Figure 5:**
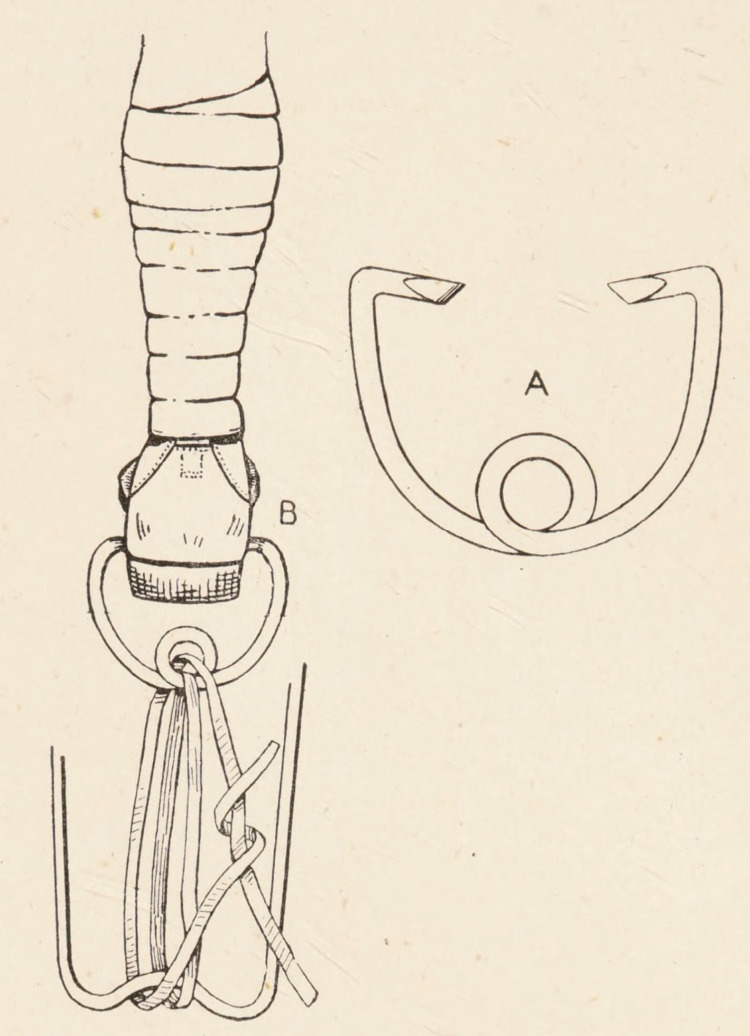
A double loop of cord creating an advantageous pulley between the boot clip and the Thomas splint to apply traction

The use of a pulley mechanism to produce traction within a Thomas splint is a re-application of a historical concept. H.M.W. Gray's text 'The Early Treatment of War Wounds' depicts a Thomas splint applied to a soldier. Looped twice between the end of the splint and the boot clip is a length of cord [2], which creates a pulley and halves the force required to attain the same traction (Figure 5).

The modernisation of the technique using a cord pulley system was described in 2014 [10]; however, it was limited to a single pulley putting additional strain on the cord.

The technique we present both simplifies and improves upon those formerly described. The disposal of the windlass removes the requirement for the two-stage tensioning required in traditional application. 

When the muscular spasm around the fracture relaxes, a re-tensioning of the traction may be required. The final knot may need to be released and the cords re-tensioned. A shoelace bow knot will allow this to be completed with ease [9]. The knot can be released whilst maintaining cord tension, further traction applied, and the knot re-tied. In a contemporary set-up, if the windlass reached its maximum twist, there would be a requirement to un-wind, re-tension, and re-wind leading to an interruption to the continuous traction and potentially causing pain. 

**Figure 6:**
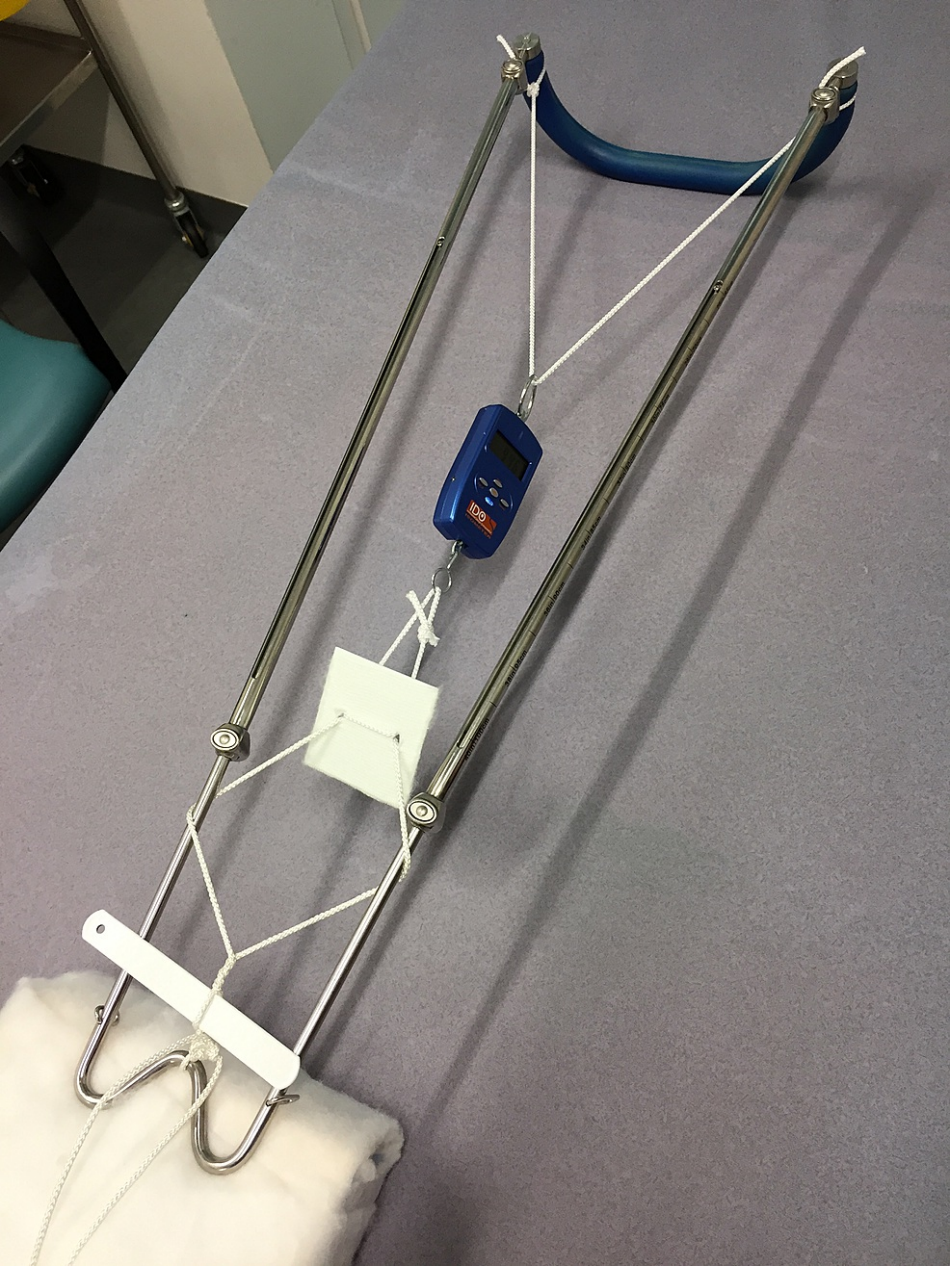
Testing the traction force that can be achieved with traditional application

We determined the traction force that could be achieved using a standard Thomas splint. A Newton meter was suspended between the proximal end of the splint and a foot plate removed from a skin traction stirrup. Traction was then set up distal to this in a traditional manner. The slack was taken up as tightly as possible and a reef knot was tied. The windlass was applied and tightened until it could be tightened no further and a reading was taken from the meter (Figure 6).

**Figure 7:**
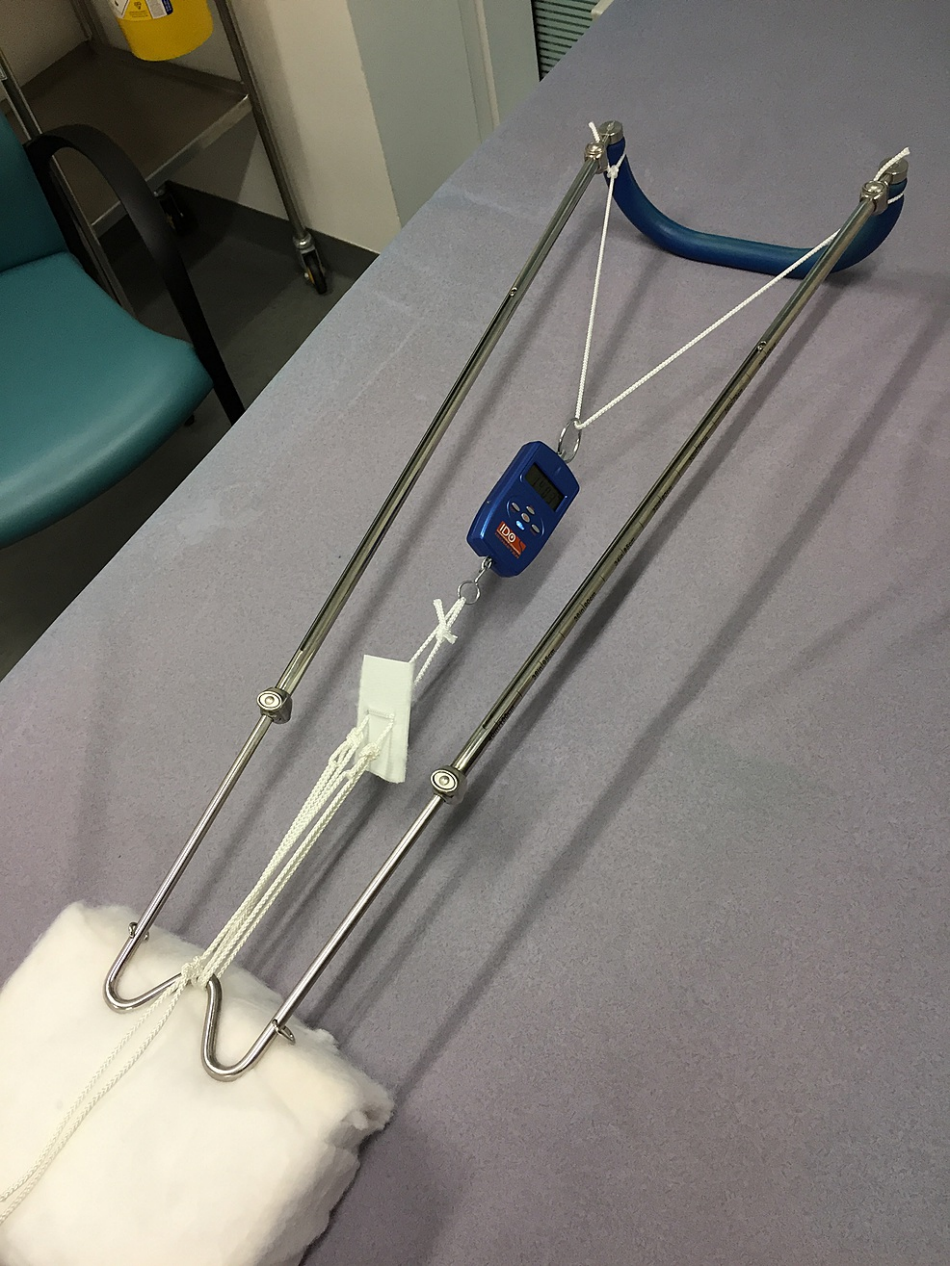
Testing the traction force that can be achieved with our method

Traction was then set up with the pulley system that we describe and a reading was taken from the meter (Figure 7).

This was repeated five times with each traction method to achieve an average.

The mean maximum force achievable by a windlass was 92 N before it became too tight to wind. The mean maximum force generated by our method that could be successfully tied off was 138 N. A weight of 7 kg is suggested as an appropriate traction weight when using traction over a pulley at the foot of the bed [11]. This will produce an approximate force of 68.6 N, which is below the force generated in both techniques tested. The practitioner applying the splint should consider that the greater the force you apply in traction, the greater the force that will be placed on the tissues of the perineum [12]. The significant complication of perineal pressure necrosis can carry a high level of morbidity and should not be allowed to occur. 

## Conclusions

After 156 years of its introduction, Thomas splint remains in frequent use throughout the world. The advantage it provides is still apparent with recent evidence suggesting that early application may reduce complications associated with the fracture. We have described a method in which the use of loops in the cord can create an advantageous pulley, thus removing the need for the windlass. We believe this method simplifies the process of applying adequate traction whilst allowing for the easy re-tensioning following muscle relaxation. 
